# Translational Studies on the Potential of a VEGF Nanoparticle-Loaded Hyaluronic Acid Hydrogel

**DOI:** 10.3390/pharmaceutics13060779

**Published:** 2021-05-22

**Authors:** Joanne O’Dwyer, Robert Murphy, Arlyng González-Vázquez, Lenka Kovarova, Martin Pravda, Vladimir Velebny, Andreas Heise, Garry P. Duffy, Sally Ann Cryan

**Affiliations:** 1 Drug Delivery & Advanced Materials Team, School of Pharmacy & Biomolecular Sciences, Royal College of Surgeons in Ireland (RCSI), Dublin 2, Ireland; joanneodwyer@rcsi.ie; 2 Tissue Engineering Research Group, Department of Anatomy & Regenerative Medicine, Royal College of Surgeons in Ireland (RCSI), Dublin 2, Ireland; agonzalez@rcsi.ie (A.G.-V.); garry.duffy@nuigalway.ie (G.P.D.); 3 Trinity Centre for Bioengineering, Trinity College Dublin (TCD), Dublin 2, Ireland; 4 Department of Chemistry, Royal College of Surgeons in Ireland (RCSI), Dublin 2, Ireland; robertdmurphy@rcsi.ie (R.M.); andreasheise@rcsi.ie (A.H.); 5 R&D Department, Contipro, Dolni Dobrouc 401, 561 02 Dolni Dobrouc, Czech Republic; Lenka.Kovarova@contipro.com (L.K.); martin.pravda@contipro.com (M.P.); Vladimir.velebny@contipro.com (V.V.); 6 Faculty of Chemistry, Institute of Physical Chemistry, Brno University of Technology, Purkynova 464/118, 612 00 Brno, Czech Republic; 7 SFI Centre for Research in Medical Devices (CURAM), National University of Ireland Galway (NUIG) and Royal College of Surgeons in Ireland (RCSI), Galway and Dublin 2, Ireland; 8 SFI Research Centre for Advanced Materials and Bioengineering Research Centre (AMBER), National University of Ireland Galway (NUIG), Royal College of Surgeons in Ireland (RCSI) and Trinity College Dublin (TCD), Dublin 2, Ireland; 9 Nursing and Health Sciences, Anatomy and Regenerative Medicine Institute (REMEDI), School of Medicine, College of Medicine, National University of Ireland Galway (NUIG), Galway, Ireland

**Keywords:** vascular endothelial growth factor nanoparticles, hyaluronic acid hydrogel, nanoparticle-loaded hydrogel, angiogenic growth factor, sustained release, catheter delivery, chick chorioallantoic membrane model, protein delivery

## Abstract

Heart failure has a five-year mortality rate approaching 50%. Inducing angiogenesis following a myocardial infarction is hypothesized to reduce cardiomyocyte death and tissue damage, thereby preventing heart failure. Herein, a novel nano-in-gel delivery system for vascular endothelial growth factor (VEGF), composed of star-shaped polyglutamic acid-VEGF nanoparticles in a tyramine-modified hyaluronic acid hydrogel (nano-VEGF-HA-TA), is investigated. The ability of the nano-VEGF-HA-TA system to induce angiogenesis is assessed in vivo using a chick chorioallantoic membrane model (CAM). The formulation is then integrated with a custom-made, clinically relevant catheter suitable for minimally invasive endocardial delivery and the effect of injection on hydrogel properties is examined. Nano-VEGF-HA-TA is biocompatible on a CAM assay and significantly improves blood vessel branching (*p* < 0.05) and number (*p* < 0.05) compared to a HA-TA hydrogel without VEGF. Nano-VEGF-HA-TA is successfully injected through a 1.2 m catheter, without blocking or breaking the catheter and releases VEGF for 42 days following injection in vitro. The released VEGF retains its bioactivity, significantly improving total tubule length on a Matrigel^®^ assay and human umbilical vein endothelial cell migration on a Transwell^®^ migration assay. This VEGF-nano in a HA-TA hydrogel delivery system is successfully integrated with an appropriate device for clinical use, demonstrates promising angiogenic properties in vivo and is suitable for further clinical translation.

## 1. Introduction

Heart failure causes severe patient morbidity and has a five year survival rate of approximately 50% [1,2,3,4,5,6,7]. The most common causative factor is ischemic heart disease. Blockage of a coronary vessel in ischemic heart disease can precipitate myocardial infarction (MI). During an MI, the volume of oxygen and nutrients delivered to cardiomyocytes is reduced, resulting in the death of approximately one billion cardiomyocytes [4,6,8,9,10]. Despite revascularization and current pharmacological therapy approaches aimed at restoring blood supply to the affected cardiac tissue, tissue damage tends to spread from the original area of infarct over time to take up more of the previously healthy tissue [11]. This persistent spread in damage causes loss of beating heart muscle and formation of akinetic, fibrotic scar tissue [12]. While initially a necessary and protective response, ultimately, this scar formation increases the risk of cardiac arrhythmias and also reduces the pumping capacity of the heart leading to heart failure.

Improving the vascularization of heart tissue following MI would improve oxygen and nutrient delivery to the cells in the affected area, thus preventing cardiomyocyte dysfunction [13,14]. Angiogenic growth factors, such as vascular endothelial growth factor (VEGF) could induce vessel sprouting and maturation, thus increasing the blood vessel density on the heart wall [15]. Initial efforts to administer VEGF following MI used direct intravenous or intracoronary injection, but these strategies were unsuccessful due to the short half-life of VEGF in vivo [16,17,18]. Sustained release of VEGF at the target site on the ventricle wall might overcome this issue. We have previously reported on the successful complexation of VEGF with a star-shaped polyglutamic acid (star-PGA) polypeptide to form nanoparticles [19]. These star-PGA-VEGF nanoparticles were then incorporated into a tyramine-modified hyaluronic acid (HA-TA) hydrogel [19]. Sustained VEGF release was obtained over 35 days, with VEGF bioactivity retained over this time period in vitro [19]. In order to progress this VEGF nano-in-gel delivery system for treatment of ischemic heart disease, more advanced in vivo studies, and integration with a device suitable for minimally invasive delivery, are critical to determine the usability of the formulation in the clinic.

Many models exist for in vivo testing of potentially angiogenic formulations, including hindlimb ischemia models, measurement of corneal angiogenesis in rabbits, MI induction in rodents and the chick chorioallantoic membrane (CAM) model [20]. In accordance with the principles of reduction, replacement and refinement for the use of animals in scientific research, the CAM model, using non-sentient chick embryos, was chosen for the initial in vivo testing of star-PGA-VEGF-HA-TA (nano-VEGF-HA-TA) [21,22]. The highly vascularized CAM functions similarly to the placenta in humans. Deoxygenated blood from the chick embryo flows through the vessels of this membrane and becomes oxygenated [23,24,25]. Thus, while the formulation is not being injected into the embryo, it is placed on the blood vessel network, exposing the whole animal to the treatment [26,27,28]. The addition of angiogenic factors to the CAM can promote more blood vessel formation, attract blood vessels in a particular direction and result in increased blood vessel branching [23]. Nano-VEGF-HA-TA was thus tested in this system to get an indication of both the biocompatibility and angiogenic effects of the formulation.

Feedback from clinicians indicated that any new agent for heart failure should be delivered in a minimally invasive manner and should be capable of delivery using a technique similar to those already used in the clinic to minimize the retraining needed. Transfemoral percutaneous coronary intervention (PCI) is a recommended cardiac reperfusion technique following MI [11]. Percutaneous delivery of nano-VEGF-HA-TA to the endocardial surface of the ventricle using the femoral artery access route may, therefore, be an appropriate technique for delivery in a clinical setting. The percutaneous route has previously been used for therapeutic delivery. A MyoStar^®^ catheter with NOGA mapping was used to deliver stem cells to the ventricle wall [29]. This percutaneous approach to therapeutic delivery was suggested to be safe both in patients with chronic ischemic heart disease and within ten days after MI [29]. A suitable medical device is required to allow such minimally invasive, percutaneous delivery of the nano-VEGF-HA-TA, taking into account its physicochemical characteristics. A catheter for delivery of nano-VEGF-HA-TA would need to facilitate homogenous mixing of crosslinkers at the tip, accurate injection volume and capability for multiple injections. Such device innovation was achieved in this project by collaboration with Boston Scientific, a world leader in devices for cardiovascular applications. The effect of catheter-delivery on the pharmaceutical and mechanical properties of hydrogels is not well documented in the literature. The mechanical properties, VEGF release and efficacy of the nano-VEGF-HA-TA may be affected by catheter delivery, and thus, this will be investigated herein.

In this manuscript, the previously reported star-PGA-VEGF-HA-TA (nano-VEGF-HA-TA) formulation will be tested in vivo on a CAM model to identify its suitability for large-scale in vivo testing. The potential for injecting the nano-VEGF-HA-TA through a clinically relevant endocardial injection catheter will be investigated as well as the effect of catheter injection on the resulting formulation.

## 2. Materials and Methods

### 2.1. Materials

Recombinant human VEGF_165_ and human VEGF ELISA kits were purchased from R & D Systems (Abingdon, UK). Bacteria-free eggs were obtained from Ovagen Ltd. (Mayo, Ireland). Float-A-Lyzers were purchased from Spectrum Labs (Amsterdam, The Netherlands). Tyramine-modified HA was obtained from Contipro (Dolni Dobrouc, Czech Republic).

The HA-TA hydrogel used is analogous to that previously described by this group with a HA molecular weight of (250–350 kDa) and 2–3% tyramine substitution [19,30]. In all cases in this study, the hydrogel used contains 1% *w*/*v* HA-TA reconstituted in phosphate buffered saline (PBS) from a freeze-dried powder. This HA-TA dispersion is filtered through a 0.2 µm filter to remove bacterial contaminants. Cross-linking of the individual HA-TA molecules is achieved using enzyme-based cross-linking facilitated by hydrogen peroxide (H_2_O_2_) and horseradish peroxidase (HRP). H_2_O_2_ is used at a concentration of 0.88 µmol/mL of HA-TA dispersion, while HRP is used at 0.24 U/mL of HA-TA dispersion. Gel formation is achieved by using a double syringe system complete with a static mixer to facilitate homogenous gel formation. This method is outlined in detail in the previous publications by O’Dwyer et al. [19,30].

The HA-TA hydrogels are used either alone (not loaded with any therapeutic), loaded with “free VEGF” (VEGF not encapsulated in a nanoparticle) or loaded with star-PGA-VEGF nanoparticles (nano-VEGF-HA-TA). The star-PGA used was synthesized aseptically, as previously described, and has a polypropyleneimine core with eight arms, each containing 40 glutamic acid residues (320 glutamic acid residues in total) and with a molecular weight of 42 kDa. Star-PGA-VEGF nanoparticles were formed as previously described using a self-assembly technique, where PBS was first added to an Eppendorf, followed by star-PGA and finally, VEGF. The components were left to complex at room temperature for five minutes [19]. In this study, the star-PGA-VEGF nanoparticles used had a star-PGA:VEGF ratio of 50:1 based on a VEGF molecular weight of 42 kDa. Star-PGA-VEGF 50:1 nanoparticles had the same characteristics as those previously described with a Z-average size of 415.5 nm, Zeta potential of −3.6 meV, polydispersity index of 0.2, encapsulation efficiency of >99.9% and a loading content of 1.9% *w*/*w* [19]. Hydrogels with VEGF (either “free” or as a nanoparticle) used for the CAM assay contained VEGF at a concentration of 500 ng/200 µL hydrogel portion. Formulation of the nanoparticle-loaded hydrogel was performed exactly as described previously [19,30]. The formulation containing 1% *w/v* HA-TA with free VEGF is referred to herein as free VEGF-HA-TA, while the formulation containing star-PGA-VEGF nanoparticles is called nano-VEGF-HA-TA.

### 2.2. Biocompatibility and Bioactivity of Nano-VEGF-HA-TA in an In Vivo CAM Model

#### 2.2.1. Incubation of Eggs for CAM Study

**Figure 1:**
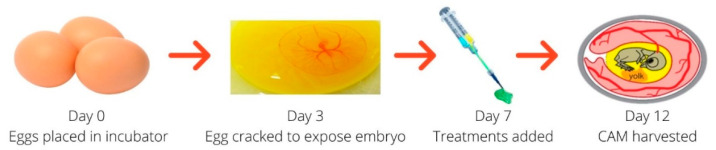
Experimental outline for the chick chorioallantoic membrane (CAM) experiment.

Fertilized, bacteria-free, GMP-compliant eggs from White Leghorn hens were purchased from Ovagen Ltd. (Mayo, Ireland). The method used was based on that used in previous publications [31,32]. The eggs were kept at 8–10 °C for three days, to prevent embryo development prior to incubation. Eggs were then placed horizontally in an incubator at 37 °C and 65% humidity for three days to facilitate embryo growth (Figure 1). On day three of incubation, the eggs were placed, one at a time, in a laminar flow hood [33]. Each egg was gently cracked into a petri dish, endeavoring not to damage the embryo. This petri dish containing the chick embryo was then placed inside a larger petri dish. PBS was placed in the larger dish, surrounding the smaller dish, to ensure a humid environment (Figure 1). The lids were then placed on both dishes and they were placed in the incubator at 37 °C. Embryo development was observed daily and any deceased embryos were removed from the incubator.

#### 2.2.2. Addition of Treatments to the CAM Membrane

Addition of treatments took place on day seven of embryo development (Figure 1). The groups tested in this study were: HA-TA alone, free VEGF-HA-TA (500 ng VEGF/200 µL gel) and nano-VEGF-HA-TA (500 ng VEGF/200 µL gel) (Table 1). This dose was chosen based on evidence in the literature of VEGF doses used in CAM studies previously [34]. One 200 µL hydrogel portion was placed, topically, on the amniotic vesicle surrounding each chicken embryo, and the petri dishes containing the embryos were closed and returned to the incubator. The treatments remained in place for five days, at which point the experiment ended (day 12 of embryo development).

#### 2.2.3. Investigation of Angiogenesis Induced on the CAM Model

Five days following treatment addition, each embryo was removed from the incubator and photographed. The position of the hydrogel scaffold was noted on each embryo and any evidence of blood cells outside the blood vessels, hyperemia, was noted. Embryos were exposed to 25 mL 10% neutral buffered formalin for two hours at room temperature, to fix them. Once fixing was complete, a region of interest around the hydrogel scaffold on each CAM was cut out and imaged using a Leica stereoscope. The region of interest was defined as a circle with a diameter of 16 mm with the hydrogel at its center. The number of vessels and the number of branch points in the photographs of the region of interest were counted manually by a researcher who did not know the identity of the samples. The length of vessels was calculated using ImageJ software (Version 1.52, National Institutes of Health, MD, USA) and the vascular length density was calculated by dividing the total blood vessel length by the area being analyzed. A distant region of interest was also identified on each CAM to compare to the vascular length density around each hydrogel. This region of interest was again a circle of 16 mm diameter at the furthest possible point from the hydrogel on the CAM.

### 2.3. Injection of Nano-VEGF-HA-TA through a Prototype AMCath Catheter

**Figure 2:**
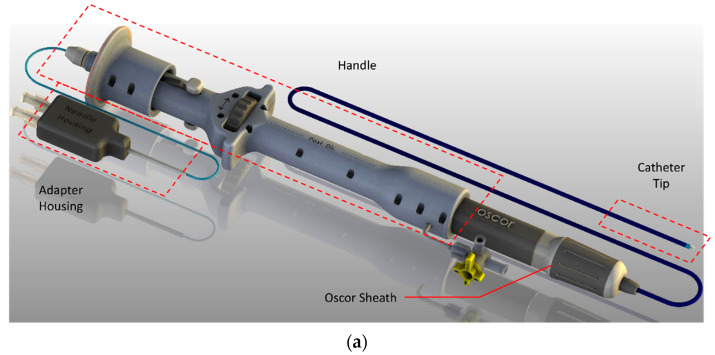

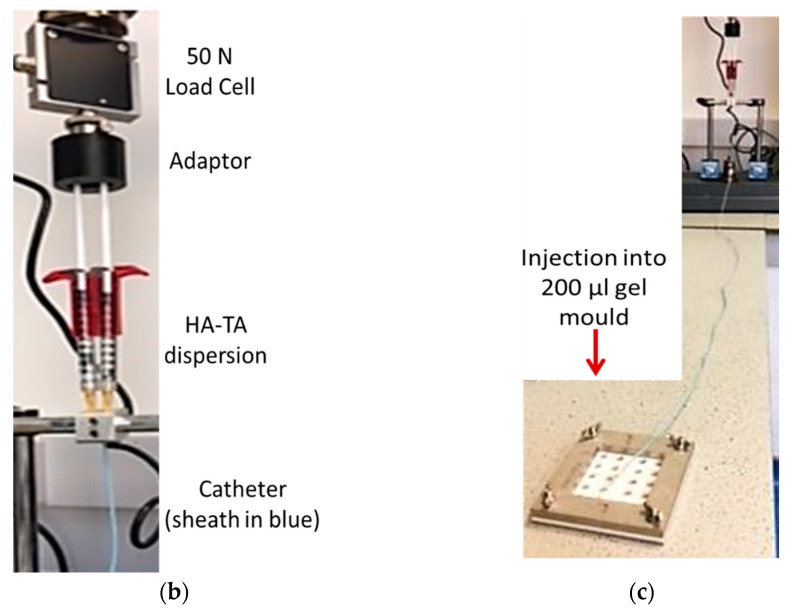
(**a**) The AMCath catheter used for hydrogel injection (Adapted from [35], SAGE Publications, 2018). (**b**) Syringes containing nano-VEGF-HA-TA attached to the prototype catheter and connected via a 3D printed adaptor to the 50 N load cell of the Zwick for testing the force required for injection. (**c**) Full length of catheter; tip is set to inject into the 200 µL injection mold (foreground).

Minimally invasive, percutaneous delivery of nano-VEGF-HA-TA in the clinic depends upon its ability to be injected through a catheter of suitable length. As a first test of the injectability of nano-VEGF-HA-TA, a prototype of the AMCath catheter, previously reported by Dolan and colleagues as suitable for delivery of a HA-TA hydrogel to the pig heart, was used (Figure 2a) [35]. Nano-VEGF-HA-TA was formulated in the usual manner and the two syringes containing the dispersion (one with HA-TA + HRP + nanoparticles and the other containing HA-TA + H_2_O_2_) were attached to the catheter. The catheter was placed in a magnetic base attached to a Zwick (Z050, Zwick/Roell, Hamburg, Germany) mechanical testing machine (Figure 2b). A 50 N load cell was used with a 3D printed adaptor connecting the catheter to the load cell of the Zwick. The machine was set to inject 200 µL over 12 s by moving at a speed of 0.5 mm per second. Injections continued until the syringes were empty or the catheter was blocked. The force was zeroed between each injection and the maximum force required for each injection was recorded. Material eluted from the catheter was placed in 200 µL molds and allowed to gel (Figure 2c).

### 2.4. Injection of Nano-VEGF-HA-TA through the AMCath Catheter with an Automated Injection System

Injection of the hydrogel into the ventricle wall will require precise control over the volume delivered in each injection. The collaborators on this project, Boston Scientific, have developed an automated injection pump that can be attached to the AMCath catheter. Addition of the injection pump allows precise control over the volume of a hydrogel delivered. The pump can be set to deliver a specific volume of hydrogel, removing issues with operator variation. Nano-VEGF-HA-TA (250 ng VEGF/mL to produce 200 µL hydrogels each containing 50 ng VEGF as used previously for in vitro work) was formulated and drawn up into two 1 mL syringes. The syringes were attached to the AMCath catheter and placed in the syringe pump. The pump was set to expel 200 µL (100 µL from each syringe) per injection. The tip of the catheter was placed in the gel mold to facilitate recovery of samples post-injection. The catheter dead volume was 600 µL, allowing seven injections of 200 µL to be performed.

#### 2.4.1. Mechanical Testing and VEGF Release of Catheter-Injected Nano-VEGF-HA-TA

Hydrogels formed following injection through the catheter connected to the syringe pump were subjected to mechanical testing with properties compared to that of hydrogels formed on injection through the benchtop hydrogel mixer.

Compression testing was carried out on a Zwick mechanical testing machine using a 5 N load cell. Samples were subjected to 20% compression at a speed of 0.01 mm/s. Young’s modulus was determined by plotting stress against strain and finding the slope of the line obtained from data between 10% and 20% compression. Hydrogels were stored in a 24-well plate in 1 mL PBS at 37 °C. The exact same procedure was then repeated on day seven.

#### 2.4.2. VEGF Release from Catheter-Injected Nano-VEGF-HA-TA

Changing the injection system may affect VEGF release from nano-VEGF-HA-TA. Nano-VEGF-HA-TA hydrogels, formed following injection through AMCath attached to the syringe pump, were placed in Spectra/Por^®^ Float-A-Lyzers with a molecular weight cut-off (MWCO) of 300 kDa. To facilitate nanoparticle release, 200 µL PBS was placed on top of the hydrogel. The Float-A-Lyzer was placed in 5 mL PBS in the receptor fluid container. At the specified time-points—8, 24, 48, 72, 96 h and days 7, 14, 21, 28, 35 and 42—the 5 mL release medium was removed and replaced with 5 mL fresh, pre-warmed PBS. The release medium was frozen for later analysis. At the conclusion of the experiment, the hydrogel samples were degraded with 1 mL of 300 IU/mL hyaluronidase, and the resulting solution as well as the release supernatant from the various time points were analyzed via ELISA to calculate the VEGF concentration.

#### 2.4.3. Biocompatibility and Bioactivity of Released VEGF

To ensure the bioactivity of the released VEGF and to ensure that no deleterious degradation components existed, a suite of in vitro biocompatibility and bioactivity assays were performed on the concentrated release supernatant from nano-VEGF-HA-TA formed on injection through the AMCath catheter. In this case, the release supernatant from all time points was pooled, concentrated using an Amicon^®^ Ultra centrifugal filter with a MWCO of 3 kDa, and used for the experiments. Fresh free VEGF at the same concentration was used as the benchmark control in all cases.

Biocompatibility and bioactivity testing was performed on human umbilical vein endothelial cells (HUVECs). HUVECs were cultured in EndoGrow cell culture medium containing all supplements except VEGF (fetal bovine serum (FBS) 2%, penicillin/streptomycin 1%, rhEGF 5 ng/mL, rhFGF 5 ng/mL, rhIGF-1 15 ng/mL, ascorbic acid 50 μg/mL, hydrocortisone hemisuccinate 1 μg/mL, heparin sulphate 0.75 U/mL and L-glutamine 10 mM). HUVECs were cultured at 37 °C and 5% CO_2_.

##### Biocompatibility

Biocompatibility testing of the pooled released supernatant was performed. Seeded in wells of a 24-well Corning^®^ Costar^®^ tissue culture plate were 3 × 10^4^ HUVECs at P4, which were given fully supplemented EndoGrow medium for 24 h to facilitate cell attachment. This medium was then removed and replaced with EndoGrow medium and the relevant treatment: 30 ng fresh free VEGF or concentrated supernatant from the nano-VEGF-HA-TA hydrogel (30 ng VEGF) formed on injection through AMCath. The medium and treatments were removed 24 h following application. 500 µL fresh EndoGrow medium and 100 µL of CellTiter 96^®^ Aqueous One Solution Cell Proliferation Assay (MTS) were added to each well. The plate was incubated for three hours at 37 °C, protected from light. Absorbance at 490 nm was then measured on a Varioskan plate reader. The metabolic activity of each treatment group was compared to that of cells not exposed to the treatment groups of fresh free VEGF or nano-VEGF-HA-TA supernatant.

##### Bioactivity—Matrigel^®^ Assay

The Matrigel^®^ assay is an in vitro indicator of the potential in vivo angiogenic effect of a formulation. HUVECs were plated onto 120 µL of growth factor reduced Matrigel^®^ in a 48-well plate at a density of 3 × 10^4^ HUVECs per well. All groups received EndoGrow cell culture medium without VEGF. The treatment groups received 30 ng of fresh free VEGF or release supernatant from nano-VEGF-HA-TA formed on injection through AMCath (30 ng VEGF). Cells were incubated at 37 °C and 5% CO_2_ and the wells were imaged at 6 and 12 h by taking five photos of each well using a Leica microscope at 10× magnification. The total tubule length per well at each time point was measured using ImageJ software.

##### Bioactivity—Scratch Assay

The scratch assay was performed to further confirm the bioactivity of the VEGF released from nano-VEGF-HA-TA, formed following injection through the 1.2 m AMCath catheter. 3 × 10^4^ HUVECs were seeded in wells of a 24-well plate and given complete EndoGrow medium. When a confluent monolayer had formed, the medium was removed and a P200 pipette tip was used to scratch a vertical line through the center of the monolayer. The wells were then washed three times with PBS to remove the cells detached by the pipette tip. EndoGrow medium without VEGF was added to all wells with either 30 ng fresh free VEGF or nano-VEGF-HA-TA supernatant containing 30 ng VEGF. The wells were imaged at 0, 6, 12 and 24 h, and the gap distance was calculated using ImageJ software.

##### Bioactivity—Transwell^®^ Migration

A Transwell^®^ migration assay was also performed to further test the bioactivity of the released VEGF. Corning^®^ Transwell^®^ hanging inserts, with a pore size of 8 μm, large enough to allow energy-dependent passage of HUVECs through the membrane, were placed in the wells of a 24-well plate. Each well contained 600 μL of serum-free medium. HUVECs at P4 or P5 were seeded on the top side of the insert membrane at a density of 3 × 10^4^ cells/insert. The plates were placed in an incubator for 2 h to facilitate cell attachment. Following this, treatments (30 ng fresh free VEGF or nano-VEGF-HA-TA supernatant with 30 ng VEGF) were placed in serum-free EndoGrow medium in wells of a fresh 24-well plate. Serum-free medium without VEGF was used as a control. The cell-coated inserts were transferred into the wells of this new plate and placed in an incubator at 37 °C and 5% CO_2_. After 24 h of incubation, the plate was removed from the incubator. The top of each insert was wiped with a cotton bud to remove any remaining HUVECs. To stain the bottom of the insert, which had been immersed in the medium or treatment, 200 μL of 2 μm calcein AM was used. The plate was incubated and protected from light at room temperature for 15 min prior to taking two representative images from the bottom of each insert. ImageJ was used to count the number of cells per image.

### 2.5. Statistical Analysis

All statistical tests were carried out on GraphPad Prism v5 (GraphPad Software Inc., Version 5, San Diego, CA, USA). Data were analyzed using a one-way ANOVA followed by a Bonferroni post-hoc test. Data are stated as mean ± standard error of the mean. The data from the CAM study represent data obtained from *n* = 5 separate embryos for each group. All in vitro data represent that obtained from three replicate samples on three individual experiments. Due to manufacturing constraints, injection of nano-VEGF-HA-TA through the AMCath connected to the syringe pump was only performed once with one catheter.

## 3. Results

### 3.1. In Vivo Testing of Nano-VEGF-HA-TA Hydrogels in a CAM Model

#### 3.1.1. Clinical Observation of Chick Embryos

Embryos were observed throughout the study and at the study endpoint. An equal number of embryos were alive in each group at the end of the study. Embryos were observed to be growing in each group and the anatomical development was normal when compared to the corresponding developmental stage in the Hamburger and Hamilton handbook [33].

#### 3.1.2. Integrity of CAM Vasculature Post-Treatment

**Figure 3:**
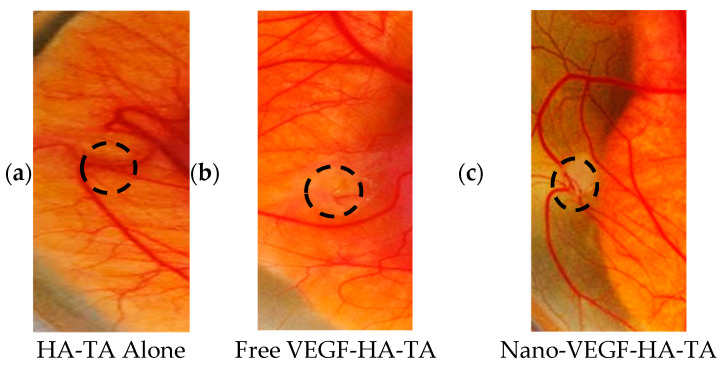
Chick chorioallantoic membrane at day twelve, exposed to (**a**) HA-TA alone, (**b**) free VEGF-HA-TA and (**c**) nano-VEGF-HA-TA. No hyperemia is present in any group. VEGF dose in all cases is 500 ng per 200 µL hydrogel portion. One representative image is shown from each group. Circle indicates hydrogel position. (*n* = 5).

The CAM model has previously been used in the literature to assess vessel permeability [36]. Hyperemia, specks of blood outside the blood vessels, signifies hyperpermeable vasculature, and therefore, compromised integrity of CAM vasculature. A representative image from each treatment group is shown in Figure 3. No evidence of hyperemia can be observed in any of the embryos, regardless of the treatment applied, and all blood is within the blood vessels, indicating the formation of blood vessels with appropriate vessel wall integrity.

#### 3.1.3. Angiogenic Effects of Nano-VEGF-HA-TA—Vessel Branching

**Figure 4:**
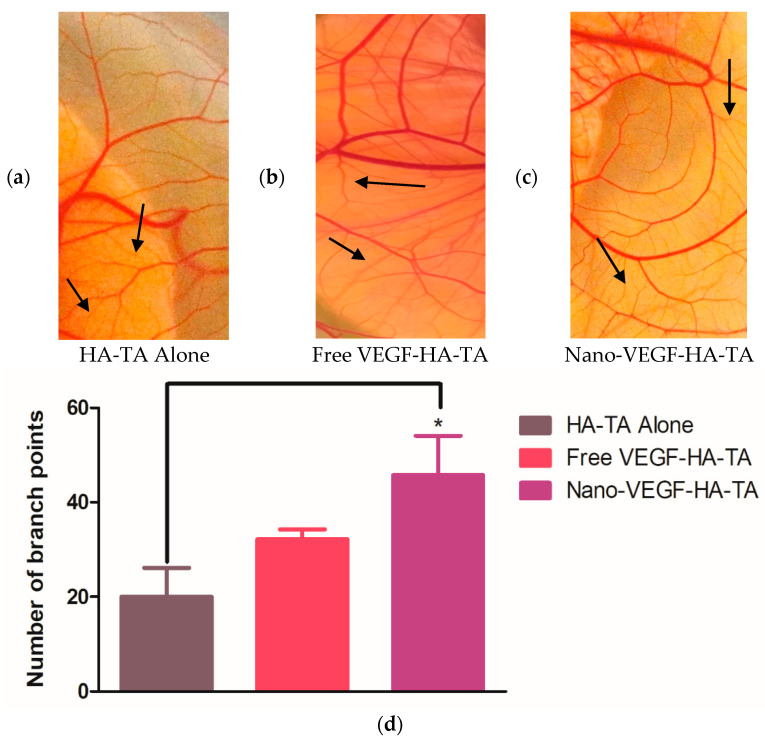
(**a**) A section of CAM from a membrane exposed to HA-TA alone. Some branch points are evident, but they are not as numerous as those observed in (**b**) a section of CAM from the free VEGF-HA-TA group or (**c**) a section of CAM from the nano-VEGF-HA-TA group. (**d**) Quantification of the number of branch points in a 16 mm region of interest surrounding the hydrogels. Free VEGF-HA-TA and nano-VEGF-HA-TA both contain 500 ng VEGF. * *p* < 0.05. (*n* = 5).

CAM studies commonly report the degree of branching as indicative of the angiogenic potential of formulations, as branching represents capillary formation. Macroscopically, a difference in the amount of branching between the control that received the HA-TA hydrogel without VEGF and the groups treated with free VEGF-HA-TA or nano-VEGF-HA-TA was evident (Figure 4a–c). This is quantified in Figure 4d for all three groups: HA-TA alone, free VEGF-HA-TA and nano-VEGF-HA-TA. Quantification took place in a region of interest surrounding the hydrogels. The hydrogel scaffolds had a diameter of 8 mm and the region of interest chosen, in all cases, was a circle around the scaffold with a 16 mm diameter, thus encompassing the hydrogel and a 4 mm radius around it in all directions. Addition of free VEGF (500 ng) to the HA-TA hydrogel resulted in a 1.61-fold, but not a statistically significant, increase in the number of branch points. Addition of VEGF nanoparticles (500 ng VEGF) to the HA-TA hydrogel (nano-VEGF-HA-TA) significantly increased the number of branch points with a 2.29-fold increase in branch points compared to HA-TA alone. Although treatment with nano-VEGF-HA-TA did increase the number of branch points, by 1.42-fold compared to treatment with free VEGF-HA-TA, this difference was not statistically significant.

#### 3.1.4. Angiogenic Effects of Nano-VEGF-HA-TA—Quantification of Vessel Formation

**Figure 5:**
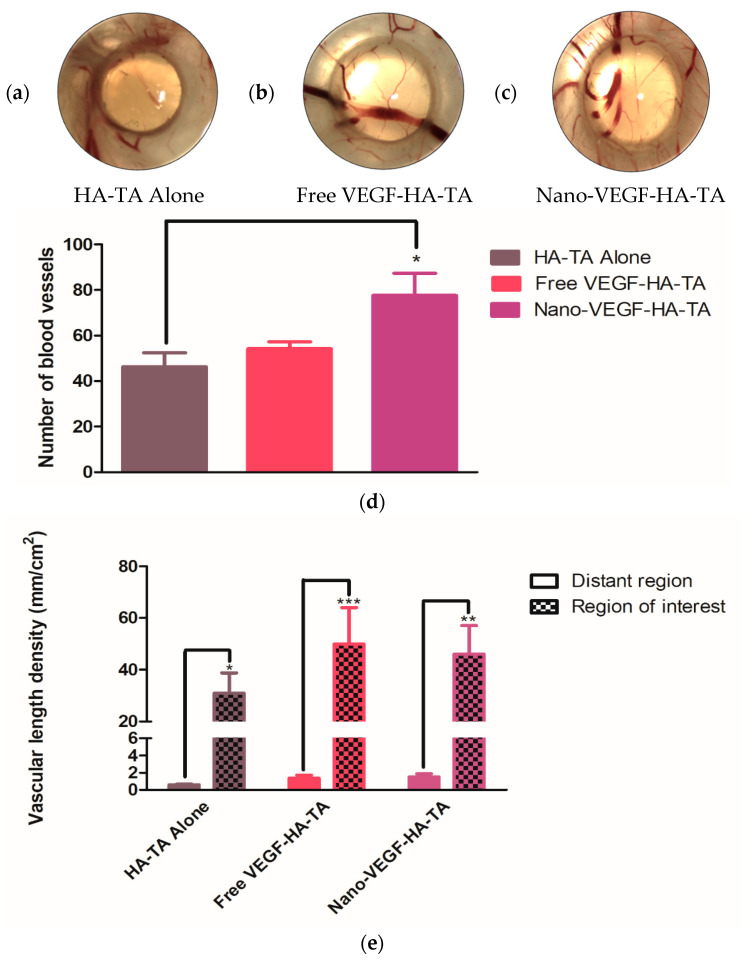
Regions of interest around (**a**) HA-TA alone, (**b**) free VEGF-HA-TA and (**c**) nano-VEGF-HA-TA, representative of those used to calculate vessel number, length density and branch points. One representative image is shown from *n* = 5 in each group. (**d**) Quantification of the number of vessels in the region of interest around HA-TA alone, free VEGF-HA-TA (500 ng VEGF) and nano-VEGF-HA-TA (500 ng VEGF). (**e**) Vascular length density in the region of interest surrounding the hydrogels compared to the vascular length density of a distant area of the chorioallantoic membrane. * *p* < 0.05, ** *p* < 0.01, *** *p* < 0.001. (*n* = 5).

Quantification of vessel number and vascular length density were used to further determine the angiogenic potential of the formulations. As outlined above, a circular region of interest with a diameter of 16 mm was identified around each hydrogel (Figure 5a–c). The number of blood vessels present in this region was counted (Figure 5d). Free VEGF-HA-TA did not significantly increase the number of vessels compared to HA-TA gel-alone treatment, with just a 1.17-fold increase in the number of vessels. However, nano-VEGF-HA-TA treatment did significantly increase the number of vessels in the region of interest compared to HA-TA gel alone, with a 1.67-fold increase in vessel number. There was no significant difference between the number of vessels formed by free VEGF-HA-TA and nano-VEGF-HA-TA, although nano-VEGF-HA-TA did increase the vessel number by 1.43-fold compared to free VEGF-HA-TA.

Figure 5e shows the vascular length density, the length of vessels normalized to the area being measured. Two separate areas were measured on each chorioallantoic membrane, one was the region of interest previously described and the other was a distant region of the membrane, away from the hydrogel, of similar area to the region of interest. Although there was no significant difference between the vascular length densities of the groups in the region of interest, free VEGF-HA-TA increased the vascular length density 1.63-fold compared to HA-TA alone, while nano-VEGF-HA-TA increased the vascular length density 1.53-fold compared to HA-TA alone. When vessel lengths are normalized to the area being measured (mm/cm^2^), the area adjacent to the gel has a significantly increased vascular length density compared to the average density for the entire CAM in all groups. In all cases, the density of vessels adjacent to the gel is increased at least 30-fold over the average vascular density at a distant point of the CAM.

### 3.2. Injection of Nano-VEGF-HA-TA through the AMCath Catheter

Initially, a prototype of the AMCath catheter was used to test whether nano-VEGF-HA-TA could be injected through a catheter, and the results of this testing are shown in Supplementary Figure S1. A catheter where the injection volume could be electronically controlled would minimize injection variability and reduce the complexity of the procedure. The AMCath catheter was, therefore, attached to a specialized syringe pump developed by collaborators Boston Scientific. This syringe pump can control injection volume, thus overcoming injection variability. Seven injections of nano-VEGF-HA-TA were successfully performed using the AMCath catheter attached to the syringe pump. At this point, the attached syringes were empty and so no further injections could be made. The catheter did not break due to excessive force during the injections. The formulations injected formed gels in the shape of the molds.

#### 3.2.1. Mechanical Testing and VEGF Release from Nano-VEGF- HA-TA Gels after Injection through the AMCath Catheter

**Figure 6:**
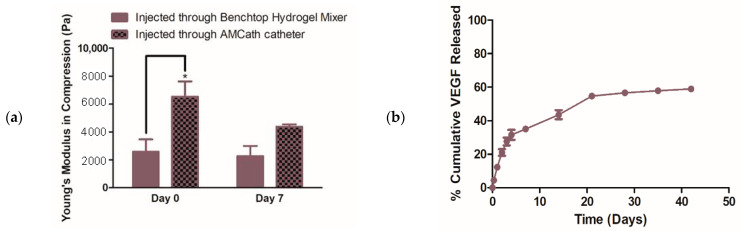
(**a**) Young’s modulus at day zero and day seven of hydrogels formed either via injection through the Benchtop Hydrogel Mixer (BHM) or via injection through AMCath connected to a syringe pump. (**b**) % cumulative VEGF release from nano-VEGF-HA-TA hydrogels formed via AMCath injection based on a dose of 50 ng VEGF per hydrogel sample. * *p* < 0.05. (*n* = four technical replicates for mechanical testing, *n* = three technical replicates for release testing).

The effect of AMCath injection on nano-VEGF-HA-TA formation was investigated using mechanical testing and a release study. Mechanical testing compared the Young’s modulus of the nano-VEGF-HA-TA (formed following injection through AMCath attached to the syringe pump) to nano-VEGF-HA-TA formed using the standard method of the benchtop hydrogel mixer. Figure 6a shows that on the day of injection through AMCath, the nano-VEGF-HA-TA hydrogel formed following injection through AMCath had a significantly higher Young’s modulus than the formulation formed using the benchtop hydrogel mixer. This significant difference was not evident at day seven post-injection.

Injecting the nano-VEGF-HA-TA dispersion through a 1.2 m catheter might change the VEGF release from the gel. VEGF release from hydrogels formed following injection through AMCath was, therefore, measured. Figure 6b shows the % cumulative release based on a VEGF dose of 50 ng per 200 µL gel. Following injection through AMCath, VEGF release from nano-VEGF-HA-TA was detected for up to 42 days. No release was detected after day 49 and so the release study was stopped at this point. Release up to day seven was rapid, with much less release occurring thereafter. Differences between the three technical replicates from day 21 onwards resulted in a standard error of the mean of less than 2% in all cases, which is not visible on the graph. VEGF release from nano-VEGF-HA-TA formed using the benchtop hydrogel mixer was shown in O’Dwyer et al. (2020) [19]. The cumulative amount released from day two onwards from the AMCath-injected hydrogel was significantly greater than that released when gel was formed using the benchtop hydrogel mixer. At the end of the experiment, hydrogels appeared intact macroscopically. The hydrogels were degraded and, on average, 3.7% of the VEGF originally loaded remained in the AMCath-formed hydrogels at this time.

#### 3.2.2. Formulation, Biocompatibility and Bioactivity Post-Injection through AMCath—Metabolic Activity, Matrigel^®^, Scratch and Transwell^®^ Migration Assays

**Figure 7:**
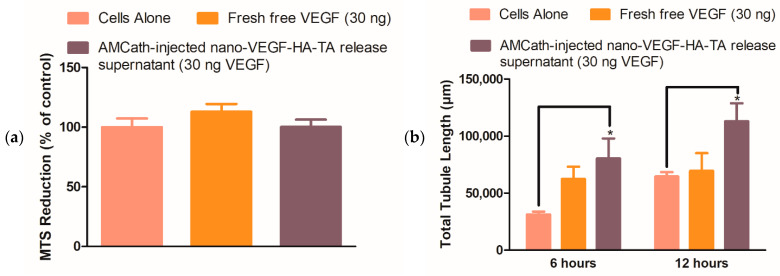

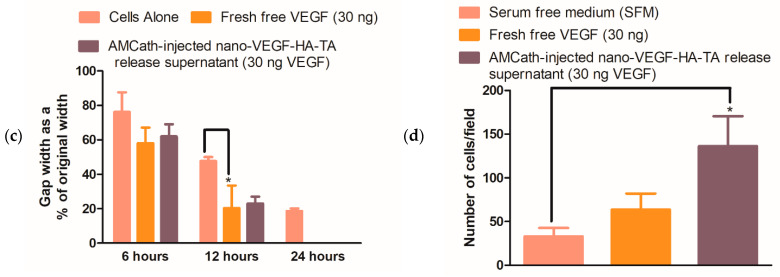
(**a**) Metabolic activity of human umbilical vein endothelial cells (HUVECs) exposed to release supernatant from AMCath-injected nano-VEGF-HA-TA, compared to cells fed with normal media without VEGF, or media containing 30 ng fresh free VEGF. (**b**) Quantified total tubule length on a Matrigel^®^ assay produced by 42-day pooled release supernatant from nano-VEGF-HA-TA formulations formed via injection through AMCath compared to the tubule length produced by untreated cells or those exposed to a similar dose (30 ng) of fresh free VEGF. (**c**) Quantification of remaining gap width on a scratch assay, where zero indicates complete gap closure. HUVECs were exposed to nano-VEGF-HA-TA release supernatant or fresh free VEGF, each containing 30 ng VEGF, and cells not treated with VEGF were used as a control. (**d**) Cell migration as determined by number of calcein stained cells per field on a Transwell^®^ migration assay. Quantification of the migration of HUVECs in medium without VEGF (cells alone in serum-free medium) is compared to that achieved by HUVECs treated with 30 ng fresh free VEGF or pooled release medium from AMCath formed nano-VEGF-HA-TA also containing 30 ng VEGF. * *p* < 0.05. (*n* = 3 technical replicates).

Previous in vitro and in vivo work has suggested that the nano-VEGF-HA-TA formulation is biocompatible. Although catheter delivery would be unlikely to affect this, biocompatibility of the formulation post-injection would still need to be confirmed prior to clinical use. Thus, metabolic activity of HUVECs exposed to the AMCath-formed hydrogel release supernatant was measured 24 h after supernatant application. Metabolic activity was compared to that of HUVECs fed with normal cell culture medium without VEGF supplementation or medium supplemented with 30 ng fresh free VEGF. No significant differences in metabolic activity were observed between the groups (Figure 7a).

It is essential that the nano-VEGF-HA-TA retain its bioactivity following injection. To ensure bioactivity was not compromised by AMCath delivery, bioactivity of nano-VEGF-HA-TA post-AMCath delivery was determined using in vitro tests relevant to angiogenesis. Three separate in vitro tests were used to determine the bioactivity of the nano-VEGF-HA-TA release supernatant. The Matrigel^®^ assay examined the ability of HUVECs to form microvessel structures, while the scratch assay and Transwell^®^ migration assay measured cell migration. In all cases, these tests used the concentrated, pooled release supernatant from the 42-day release study performed on the nano-VEGF-HA-TA post-AMCath injection using fresh, unformulated (fresh free) VEGF as a control.

Total tubule length on the Matrigel^®^ assay was significantly increased in the presence of the AMCath release supernatant compared to the untreated controls at both 6 and 12 h (Figure 7b). In addition, the VEGF released from nano-VEGF-HA-TA produced a 1.3-fold and 1.62-fold increase in total tubule length compared to fresh free VEGF at 6 and 12 h, respectively.

Figure 7c shows the reduction in gap width on a scratch assay induced by the AMCath-formed nano-VEGF-HA-TA hydrogel release supernatant, fresh free VEGF or untreated cells. At 24 h, both the fresh free VEGF and the AMCath release supernatant were capable of closing the formed gap, while the cells not exposed to VEGF were not.

As a final test of the VEGF bioactivity, a Transwell^®^ migration assay was undertaken. The release medium from the nano-VEGF-HA-TA post-AMCath injection induced significantly more cell migration than cells alone (Figure 7d and Supplementary Figure S2). More cells also migrated in the nano-VEGF-HA-TA release medium treated group than in the group treated with fresh free VEGF, although the difference here was not significant.

## 4. Discussion

Herein, a number of key studies for the translation of a novel nano-in-gel delivery system for the targeted delivery of VEGF were conducted. A CAM study was performed as the initial in vivo interrogation of the nano-VEGF-HA-TA formulation. The first output from the CAM study was the observed lack of toxicity of the delivery system. It has previously been suggested that a mortality rate of 50% is to be expected with the CAM model; a 40% mortality rate was observed in all groups here, slightly better than that cited in the literature [37]. All chick embryos displayed common anatomical features; eyes, legs and wings were visible in all cases, further signaling a lack of toxicity. While this would need further investigation in other animal models, the absence of evidence of toxicity at this early stage is promising.

Administration of VEGF without other growth factors has been reported to produce immature, hyperpermeable blood vessels, although this finding was related primarily to the delivery of nucleic acids encoding for VEGF [38]. Increased vessel permeability has previously been observed in CAM assays by Dunn et al. and is evidenced by the presence of hyperemic foci, essentially specks of blood in the space between vessels [36]. No hyperemia was evident in the CAM study performed herein, which corroborates evidence in the literature that the controlled delivery of the VEGF protein overcomes this hyperpermeability issue [38].

Having determined the health of the embryos and the integrity of the blood vessels formed, the angiogenic properties of the formulations were examined. Branching of vessels is a key process in the development of an interlinked vessel network and is a commonly reported output of the chick CAM experiment post-treatment with angiogenic factors. In Figure 4, an area of CAM treated with (a) HA-TA alone was compared to that exposed to (b) free VEGF-HA-TA and (c) nano-VEGF-HA-TA. The most branching points and capillary-like structures were visible following treatment with nano-VEGF-HA-TA, and this is quantified in Figure 4d. While free VEGF-HA-TA did not significantly increase the number of branch points compared to HA-TA alone, nano-VEGF-HA-TA did significantly (*p* < 0.05) increase the number of branch points compared to treatment with the HA-TA gel alone. This indicates that the nano-VEGF formulation could be protecting VEGF from degradation, allowing more bioactive VEGF to act on the blood vessels. Differences in VEGF release from the formulations have previously been shown with 17% of the loaded VEGF detected over 42 days from HA-TA gel alone, compared to 40% of loaded VEGF released from nano-VEGF-HA-TA (nano-in-gel) [19]. The combination of improved stability and release of VEGF may explain the difference in branch point number observed here between the treatment groups.

A similar trend occurred with the number of blood vessels observed in the model post-treatment. Figure 5d demonstrated that nano-VEGF-HA-TA treatment significantly increased the number of vessels in the region of interest compared to HA-TA gel-alone treatment, while free VEGF-HA-TA did not. Previous reports in the literature suggest that 50–80 vessels are commonly seen in this model when treated with angiogenic formulations [39]. The HA-TA gel-alone formulation had, on average, 46 vessels in the region of interest, close to the range for angiogenic formulations. This is not surprising as the angiogenic effects of HA are well documented. Nano-VEGF-HA-TA had an average of 92.3 vessels in the region of interest, indicating its angiogenic potential. The free VEGF-HA-TA treatment group had an average of 54 vessels in the region of interest. This was not significantly more than the number of vessels in the HA-TA gel-alone treatment group. The authors again hypothesize that this is due to the short half-life of released VEGF (approximately 40 min in vivo) [16]. In comparison, VEGF released from nano-VEGF-HA-TA formulations may have improved stability due to binding of the VEGF to the star-PGA. The difference in VEGF release between free VEGF-HA-TA and nano-VEGF-HA-TA, as discussed above (17% VEGF release from free VEGF-HA-TA formulations over 42 days compared to 45% release from nano-VEGF-HA-TA formulations), may also account for the absence of a significant effect with free VEGF-HA-TA treatment [19].

In considering the clinical application of this formulation, the total vessel length is important. A longer interlinked vessel network will supply blood to more cells. The planned injection protocol would be to inject portions of the hydrogel at areas around the infarcted zone. Thus, the vascular length density was measured and the region of interest was compared to a distant area of the CAM for all formulations. All three formulations—HA-TA gel alone, free VEGF-HA-TA and nano-VEGF-HA-TA—significantly increased the vascular length density in the region of interest surrounding the hydrogel, compared to a distant area of the CAM. In all cases, this increase was in the region of 30-fold for all three groups. This indicates the angiogenic potential of HA-TA itself and the formulations being tested. Taken together, the results from the CAM assay indicate a lack of apparent toxicity issues, an absence of leaky blood vessels, the angiogenic potential of the formulations being tested and that the nano-VEGF-HA-TA may, through its release characteristics and/or protection of the VEGF, provide advantages over free VEGF-HA-TA. There are limitations to this study in that it does not involve the administration of the formulation to the heart and does not use a model of myocardial infarction. Toxicity issues may also be different in a different species. However, the promising bioactivity and biocompatibility results justified testing whether nano-VEGF-HA-TA would be suitable for administration in a clinical setting. This is a critical point to investigate before progressing the formulation to further, whole animal, in vivo testing.

The transfemoral percutaneous route of delivery had been identified as a potential means of administration for this formulation in vivo and would involve inserting a catheter into the femoral artery in the groin and progressing it through this artery all the way to the left ventricle. This technique would be similar to percutaneous interventions currently performed and so would not require extensive retraining of clinicians. The total length of catheter required would be 1.2 m. To allow injection of the material into various regions of the ventricle wall, the surgeon must be able to push the gel out of the catheter. Initial testing was undertaken using a prototype of the AMCath catheter, which has successfully been used for endocardial injection of stem cells in a porcine model [35]. The data, shown in the supplementary material (Supplementary Figure S1), shows that nano-VEGF-HA-TA can be injected through the catheter without blocking or breaking the catheter, and that gelation can occur following catheter injection.

However, the optimal catheter for delivery of nano-VEGF-HA-TA would additionally need to facilitate accurate injection volume over multiple injections. Alterations in the pressure applied to a catheter between various injections by the operator have previously been suggested to change the depth of hydrogel injection into the ventricle wall [40]. Therefore, a device that could automatically deliver a predefined quantity of formulation, e.g., a catheter device with an automated injection system, would reduce the variability and complexity of the final clinical procedure. Such a device was developed by collaborators Boston Scientific. This system allows a specific volume of a formulation to be delivered by setting a specific injection volume using a mechanical device attached to the syringes of the catheter. In this work, an injection volume of 200 µL was set as this has previously been used for the delivery of an IGF-1 and HGF containing ureido-pyrimidinone hydrogel to the ventricle wall in a porcine model of MI [41].

Nano-VEGF-HA-TA was successfully injected through the AMCath catheter when connected to the syringe pump without breaking the catheter, and seven injections were possible with no blockage of the catheter. Multiple injections were made to ensure the experiment was representative of previous clinical studies, e.g., the Algisyl^®^ hydrogel in clinical trials [42]. Injected dispersions formed hydrogels following injection. To prevent uncontrolled diffusion of the VEGF nanomedicines upon reaching the target site in vivo, rapid gelation following catheter injection is a key characteristic of any possible formulation for this application [41,43,44]. Gelation of the polymer dispersions within 15 s of injection is, therefore, highly advantageous.

The effect of catheter injection on the Young’s modulus of the formulation was measured. While the Young’s modulus of the catheter-injected hydrogel was significantly increased compared to a gel formed using the normal system (benchtop hydrogel mixer) at day zero, this effect had dissipated by day seven. This difference may be due to slightly different mixing when going through the catheter compared to the benchtop mixer. Of the other groups who have reported hydrogel injection through such a catheter, none have reported the effect of this on mechanical properties, to our knowledge. However, these results suggest nano-VEGF-HA-TA can be delivered through a clinically relevant catheter, multiple injections can be performed without catheter breakage or blockage and the formulation can form a gel following injection. The final step was to determine the effect of catheter injection on the release properties and bioactivity of the VEGF formulation.

Growth factor release and bioactivity can be highly sensitive to the surrounding environment, including packaging, storage conditions and interaction with delivery devices [45]. Travelling through a 1.2 m catheter may affect the rate of therapeutic cargo release and/or its integrity [40]. Therefore, the rate of VEGF release and its bioactivity post-release from the nano-VEGF-HA-TA were both assessed post-AMCath delivery and compared with the same system formed via the benchtop hydrogel mixer. Greater burst release and total release of VEGF was observed from nano-VEGF-HA-TA formulations formed on injection through AMCath compared to the same formulation prepared using the benchtop hydrogel mixer. Retention of the sustained release characteristic following injection is important to facilitate spatiotemporal control of growth factor delivery [46]. The release pattern obtained is still similar to that obtained and determined to be optimal by Silva et al. [47]. The small amount of VEGF (3.7%) recovered from the degraded hydrogels means that almost all of the loaded VEGF is capable of getting out of the hydrogel. Adding together the released VEGF and that recovered from the AMCath injected hydrogel following degradation, 64% of the loaded VEGF is accounted for. Considering the suggested 90 min half-life of VEGF in the absence of biological substances, it is reasonable to suggest that the remaining VEGF has degraded over the time course of the experiment. While Minguell et al. have found that, in the case of stem cell injection through a 1.2 m catheter, 10% of cells are lost, no groups have previously looked at protein loss on catheter injection [48].

Testing of the biocompatibility of the AMCath injected hydrogel indicated a similar lack of toxicity to that seen in previous in vitro work and on the CAM assay. Matrigel^®^, Scratch and Transwell^®^ migration assays determined that the bioactivity of VEGF within the nano-VEGF-HA-TA formulation was maintained on injection through AMCath and subsequent release. The preservation of VEGF bioactivity following injection of nano-VEGF-HA-TA through AMCath and subsequent release is an important scientific and clinical finding for the field. Concerns have previously been raised about the stability of proteins in controlled release systems [44,49]. The specific 3D structure of proteins is highly sensitive to changes in temperature, shear and ionic strength, thus maintaining protein bioactivity during injection and a period of prolonged release could be difficult [49,50]. Again no relevant literature exists on the effect of catheter injection on the bioactivity of delicate proteins in hydrogels. In the case of stem cells, a 3% reduction in cell viability was observed following catheter injection, suggesting that the injection forces may have some effect on sensitive cargo [48]. The preserved bioactivity of VEGF following injection of nano-VEGF-HA-TA through AMCath in this manuscript is, therefore, an important scientific finding more broadly for the field of integrated drug-device delivery systems.

## 5. Conclusions

Improving angiogenesis following an MI has been proposed as a potential step to prevent heart failure and its associated adverse sequelae. Achieving sustained release of an angiogenic growth factor in the heart remains elusive. Herein, a nano-VEGF-HA-TA formulation was investigated that may overcome issues with previous formulations. Using an in vivo CAM assay, the nano-VEGF-HA-TA formulation was found to be biocompatible and significantly improved a number of angiogenic end points compared to treatment with a HA-TA hydrogel alone. Nano-VEGF-HA-TA was successfully injected through a clinically relevant catheter for endocardial delivery and retained its release properties and its bioactivity, as determined in a number of in vitro assays. Nano-VEGF-HA-TA has shown its potential for clinical translation and will now progress to large-scale in vivo studies, and more broadly, provides a platform for the minimally invasive delivery of a controlled release delivery system for proteins to the myocardium.

## 6. Patents

Some of the work contained herein is covered under patent application number 1821014.6.

## Figures and Tables

**Table 1:** Formulations tested in the chick chorioallantoic membrane study and the amounts of star-polyglutamic acid (PGA) or vascular endothelial growth factor (VEGF) per 200 µL gel portion for each. Free VEGF refers to the VEGF in the HA-TA hydrogel that is not encapsulated in a nanoparticle.

Formulation	Description	VEGF	Star-PGA
HA-TA alone	1% HA-TA alone	-	-
Free VEGF-HA-TA	1% HA-TA + free VEGF	500 ng	-
Nano-VEGF-HA-TA	1% HA-TA + star-PGA-VEGF 50:1 nanoparticles	500 ng	25 µg

## Data Availability

The data presented in this study are available on request from the corresponding author.

## Supplementary Materials

The following are available online at https://www.mdpi.com/article/10.3390/pharmaceutics13060779/s1: Figure S1. Injection curves for nano-VEGF-HA-TA injected through a 1.2 m catheter.; Figure S2. Migration of Human umbilical vein endothelial cells (HUVECs) on a Transwell^®^ migration assay. Migration of HUVECs in medium without VEGF (cells alone in serum free medium) is compared to that achieved by HUVECs treated with 30 ng fresh VEGF or pooled release medium from AMCath formed nano-VEGF-HA-TA also containing 30 ng VEGF. (*n* = 3 technical replicates).
