# Supplementary material for: Translational Studies on the Potential of a VEGF Nanoparticle-Loaded Hyaluronic Acid Hydrogel
Source: Pharmaceutics. 2021 May 22;13(6):779. doi: 10.3390/pharmaceutics13060779 (PMC8224549; doi:10.3390/pharmaceutics13060779)
Supplement: Supplementary file 1 [file pharmaceutics-13-00779-s001.zip › pharmaceutics-1208847-supplementary.pdf]

pharmaceutics

Supplementary Materials: Translational Studies on the Potential of a VEGF Nanoparticle-Loaded Hyaluronic Acid Hydrogel

Joanne O'Dwyer, Robert Murphy, Arlyng González-Vázquez, Lenka Kovarova, Martin Pravda, Vladimir Velebny, Andreas Heise, Garry P. Duffy and Sally Ann Cryan

Viability of injecting nano-VEGF-HA-TA through a prototype AMCath catheter

Nano-VEGF-HA-TA was injected through a prototype of the AMCath catheter, previously reported by Dolan and colleagues as suitable for delivery of a HA-TA hydrogel to the pig heart.

Nano-VEGF-HA-TA was formulated and the two syringes containing the dispersion were attached to the catheter. The catheter was placed in a magnetic base attached to a Zwick (Z050, Zwick/Roell, Germany) mechanical testing machine. A 50 N load cell was used with a 3D printed adaptor connecting the catheter to the load cell of the Zwick. Each injection was performed over 12 seconds, in which time 200 µl of the formulation was expelled from the catheter (injection rate 1 ml/minute). The force was zeroed between each injection and the maximum force required for each injection was recorded.

The maximum force required to inject nano-VEGF-HA-TA through the AMCath prototype catheter was 7.75 N. This force is lower than that which can be exerted by human syringe operators (50–70 N) [7]. Figure S1 shows the individual injection curves obtained for the formulation. Six injections of the nano-VEGF-HA-TA formulation were possible without any change in the force required or blockage of the catheter.

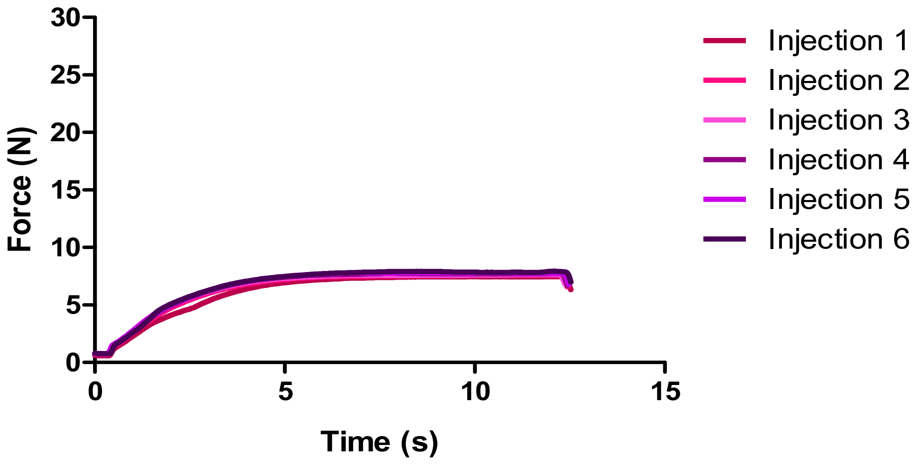

Figure S1. Injection curves for nano-VEGF-HA-TA injected through a 1.2 m catheter.

Cell migration in the presence of AMCATH-injected nano-VEGF-HA-TA

Cell migration was measured on a Transwell® migration assay in response to the release supernatant from AMCATH injected nano-VEGF-HA-TA. Human umbilical vein endothelial cells (HUVECs) in serum-free medium and HUVECs in the presence of fresh free VEGF (30 ng) were used as controls.

(a)

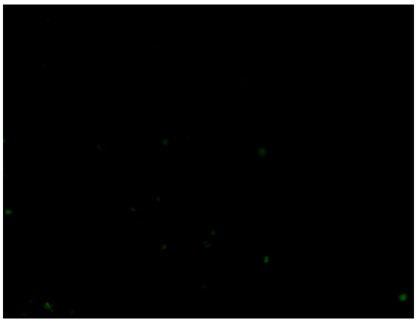

Serum Free Medium

(b)

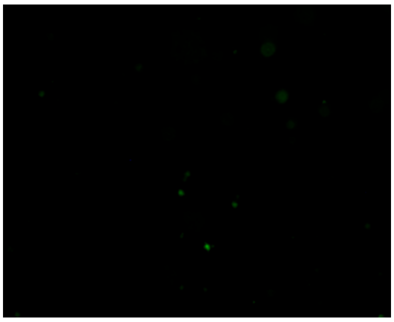

Fresh free VEGF (30 ng)

(c)

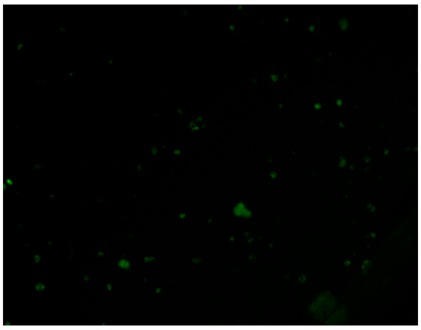

AMCATH-injected nano-VEGF-HA-TA release supernatant (30 ng VEGF)

Figure S2. Migration of Human umbilical vein endothelial cells (HUVECs) on a Transwell® migration assay. Migration of HUVECs in medium without VEGF (cells alone in serum free medium) is compared to that achieved by HUVECs treated with 30 ng fresh VEGF or pooled release medium from AMCath formed nano-VEGF-HA-TA also containing 30 ng VEGF. (n = 3 technical replicates).
